# Immunotherapy and Radioimmunotherapy for Desmoplastic Small Round Cell Tumor

**DOI:** 10.3389/fonc.2021.772862

**Published:** 2021-11-19

**Authors:** Madelyn Espinosa-Cotton, Nai-Kong V. Cheung

**Affiliations:** Department of Pediatrics, Memorial Sloan Kettering Cancer Center, New York, NY, United States

**Keywords:** DSRCT = desmoplastic small round cell tumor, antibodies, immunotherapy, targeted therapy, radioimmunotherapy, CAR T cell

## Abstract

Desmoplastic small round cell tumor (DRSCT) is a highly aggressive primitive sarcoma that primarily affects adolescent and young adult males. The 5-year survival rate is 15-30% and few curative treatment options exist. Although there is no standard treatment for DSRCT, patients are most often treated with a combination of aggressive chemotherapy, radiation, and surgery. Targeted therapy inhibitors of PDGFA and IGF-1R, which are almost uniformly overexpressed in DSRCT, have largely failed in clinical trials. As in cancer in general, interest in immunotherapy to treat DSRCT has increased in recent years. To that end, several types of immunotherapy are now being tested clinically, including monoclonal antibodies, radionuclide-conjugated antibodies, chimeric antigen receptor T cells, checkpoint inhibitors, and bispecific antibodies (BsAbs). These types of therapies may be particularly useful in DSRCT, which is frequently characterized by widespread intraperitoneal implants, which are difficult to completely remove surgically and are the frequent cause of relapse. Successful treatment with immunotherapy or radioimmunotherapy following debulking surgery could eradiate these micrometasteses and prevent relapse. Although there has been limited success to date for immunotherapy in pediatric solid tumors, the significant improvements in survival seen in the treatment of other pediatric solid tumors, such as metastatic neuroblastoma and its CNS spread, suggest a potential of immunotherapy and specifically compartmental immunotherapy in DSRCT.

## Introduction

### Background

Desmoplastic small round cell tumor (DRSCT) was first described in 1989 by Gerald and Rosai as a highly aggressive primitive sarcoma characterized by nests of blue-staining tumor cells surrounded by dense stroma. Of note was the fact that these tumor cells were positive for markers of mesenchymal, neural, and epithelial lineages, suggesting that they may arise from undifferentiated progenitor cells (1, 2). A (11:22), (p13:q12) chromosomal translocation is present in all cases, resulting in an EWS-WT1 gene fusion product (3–5). This feature is considered pathognomonic and is required for its definitive diagnosis (6). DSRCT is rare, with an age-adjusted incidence rate of 0.3/million in the United States (7), and primarily arises in adolescent and young adult males, with around 80% of patients being male and an average age of 18-22 years at diagnosis (2, 8). It may disproportionately affect African Americans (7, 9). Overall 5-year survival is a dismal 15-30% (7, 10).

### Clinical Presentation and Staging

Most patients initially present with abdominal pain and distension, evidence of ascites caused by extensive tumor seeding of the peritoneum (6). The vast majority (>90%) of primary tumors are found on the serosal surfaces of the abdomen, but others have been found in the thoracic cavity, the skull, or even the hand (8). Rarely, early-stage DSRCT is discovered incidentally during surgery or imaging for other indications. More commonly, however, the disease has spread extensively and is considered Stage IV at the time of diagnosis. Because of this, a new staging system for DSRCT has been proposed by Hayes-Jordan and colleagues: Stage I would include patients with one or two abdominal tumors, Stage II would include patients with extensive peritoneal spread, Stage III would include patients with peritoneal disease plus liver metastases, and Stage IV would include patients with disease that has spread outside the abdomen (6).

### Treatment

There is no standard therapy for DSRCT. Treatment typically consists of multimodal neoadjuvant chemotherapy followed by surgery and radiation. Treatment with the P6 protocol – cyclophosphamide, doxorubicin and vincristine alternating with ifosfamide and etoposide – is used in the United States, whereas a slightly different protocol used to treat Ewing Sarcoma patients (vincristine, doxorubicin, and cyclophosphamide alternating with ifosfamide and etoposide) is the most common regimen in other parts of the world (11, 12). Because of the extent of disease spread within the peritoneal cavity, surgery is most effective after neoadjuvant chemotherapy, since these tumors are typically responsive to chemotherapy and either shrink or become less vascularized (6). Complete surgical resection of all visible tumors was found to be essential for survival beyond 3 years, with 58% of patients who underwent complete resection surviving to 3 years compared to 0% of patients whose tumors were not resected and instead received only chemotherapy and radiation (10). While surgical excision of all visible tumors is critical, it may be impossible to remove every tumor cell from the peritoneal cavity. Hyperthermic intraperitoneal chemotherapy (HIPEC) has been proposed as a treatment for microscopic residual disease after complete surgical resection. A recent phase II clinical trial showed that HIPEC using cisplatin is effective at improving survival, with a 3-year overall survival rate of 79% (13). However, this treatment modality does not appear effective for patients whose tumors were not able to be completely removed during surgery (14). Whole abdominopelvic irradiation has also been used to treat residual disease following surgery and appears to reduce the incidence of peritoneal relapse, however, severe gastrointestinal and hematopoietic toxicity is common (15, 16). Intensity modulated radiation therapy reduces grade 2-4 toxicities without compromising efficacy (17). Autologous stem cell transplant has been investigated and does not appear to improve outcomes for DSRCT patients (18); allogenic stem cell transplant could be an alternative (19). Several clinical trials are currently underway to test new combinations of chemotherapy and targeted therapy (**Table 1**). Progress has been made in extending survival, however, cures are rare.

**Table 1:** Current clinical trials using chemotherapy and targeted therapy for DSRCT.

Trial	Phase	Therapy	Status	Study term	Actual Enrollment
NCT01189643	Early 1	Irinotecan, temozolomide and bevacizumab in combination with existing high dose alkylator based chemotherapy	Active, not recruiting	08/2010 – 08/2022	15
NCT03478462	1	CLR 131 (phosopholipid drug conjugate)	Recruiting	04/2019 – 12/2024	30*
NCT03600649	1	Seclidemstat (LSD1 inhibitor)	Recruiting	06/2018 – 12/2021	50*
NCT04145349	1/2	Ramucirumab, cyclophosphamide, vinorelbine	Recruiting	01/2020 – 01/2024	34*
NCT04095221	1/2	Prexasertib, irinotecan, temozolomide	Recruiting	09/2019 – 09/2022	30*
NCT04901806	1/2	PBI-200 (TRK inhibitor)	Recruiting	07/2021 – 06/2024	74*
NCT01946529	2	Vincristine, doxorubicin, cyclophosphamide, ifosfamide, etoposide, temozolomide, temsirolimus, bevacizumab, sorafenib, surgery, and radiation	Active, not recruiting	12/2013 – 07/2026	24
NCT03275818	2	Nab-paclitaxel	Active, not recruiting	05/2017 – 05/2021	60*

*For ongoing studies the estimated enrollment is provided in lieu of actual enrollment.

## Mutations, Targets, and Dysregulated Pathways

Many common mutations have been found in DSRCT (**Table 2**), some of which have been explored as targets for therapy. Several of these are targets of the EWS-WT1 fusion protein, which is present in every case of DSRCT and is required for positive diagnosis (6). This protein results from a chromosomal translocation involving breakage of chromosomes 11 and 22 at sites of genes known to be involved in Wilms’ tumor (*WT1*) and Ewing’s sarcoma (*EWSR1*), respectively (20). *WT1* is a tumor suppressor gene encoding a transcription factor (WT1) that generally represses gene expression and was first noted for its deletion in Wilms’ tumor (21, 22). Fusion of the transcription-activating N-terminal domain of *EWS* to a set of zinc fingers in *WT1* produces a unique transcription factor capable of upregulating a number of genes that promote tumor progression, many of which are repressed by wild-type WT1 (20).

**Table 2:** Mutations and dysregulated pathways in DSRCT.

Mutation/pathway	Source material	Publication year	PMID
Acetylcholine receptor (AChR)	2 tumor specimens	2008	18568996
Androgen receptor (AR)	27 tumor specimens	2007	16896931
	7 tumor specimens	2017	28415643
BAIAP3 promoter	2 tumor specimens	2002	12498718
B7-H3	37 tumor specimens	2001	11358824
Connective tissue growth factor (CCN2)	3 tumor specimens	2004	15047749
c-Kit	27 tumor specimens	2007	16896931
DNA damage-response pathway	7 tumor specimens	2018	30486883
	2 PDX models	2019	30563935
Epidermal growth factor receptor (EGFR)	12 tumor specimens	2015	25906748
EMT/MET	7 tumor specimens	2018	30486883
	7 tumor specimens	2017	28415643
Equilibrative nucleotide transporter 4 (ENT4)	4 tumor specimens	2008	18523561
EWS-WT1	5 tumor specimens	1994	8187063
GD2	20 tumor specimens	2016	27304202
HER2	1 patient	2015	25800760
	23 patient specimens	2003	12640103
IL-2/15Rbeta	16 tumor specimens	2002	11960373
Insulin growth factor receptor I (IGF-1R)	2 EWS/WT1-transduced osteosarcoma cell lines	1996	8702614
LRRC15	8 tumor specimens	2003	12923058
PI3K/Akt/mTOR	1 tumor specimen	2013	23922674
	10 tumor specimens	2014	25119929
PDGFA	5 tumor specimens	1997	9354795
MET	10 tumor specimens	2014	25119929
NTRK3	2 cell lines, 2 tumor specimens, 3 PDX models	2020	33229458
TGFbeta	10 tumor specimens	1999	10074970

### Platelet-Derived Growth Factor A

One of the most well-characterized gene targets of EWS-WT1 is *PDGFA*, whose role in DSRCT was first described by Haber and colleagues in 1997, just a few years after the identification of DSRCT as a distinct malignancy (23). This group found that *PDGFA* was upregulated in an osteosarcoma cell line following induced expression of EWS-WT1 (23). Additionally, *PDGFA* expression was found in 13/14 DSRCT tumor specimens and correlated with expression of EWS-WT1 (23). These results and subsequent publications (24, 25) confirming the role of *PDGFA* in DSRCT laid the groundwork for clinical trials using imatinib mesylate, a tyrosine kinase inhibitor that targets *abl*, c-Kit, and PDGF receptor (PDGFR) and is known for its use in the treatment of chronic myelogenous leukemia (26). Unfortunately none of these trials have shown any efficacy in DSRCT (27–29).

### Insulin-Like Growth Factor 1 Receptor (IGF-1R)

Another target of EWS-WT1 is IGF-1R, a tyrosine kinase receptor that is frequently upregulated in cancer cells, leading to dysregulation of the IGF pathway (30). There are several reports of DSRCT patients presenting with severe hypoglycemia as a result of an elevated IGF-II : IGF-I ratio, a consequence of IGF pathway dysregulation (31, 32). Several IGF inhibitors have been tested in DSRCT patients in early phase clinical trials. In a phase II trial of 16 DSRCT patients treated with ganitumab [monoclonal antibody (mAb) IGF-1R inhibitor], 25% (4/16) achieved clinical benefit (PR + SD ≥ 24 weeks) (33). In another study combining cixutumumab (mAb IGF-1R inhibitor) and temsirolimus (mTOR inhibitor) in 20 patients with Ewing’s sarcoma family tumors, two DSRCT patients achieved partial tumor regression and one progressed (34).

### Androgen Receptor (AR)

Because DSRCT predominantly affects male patients, it has been hypothesized that androgen receptor (AR) could play a role in this disease. In one study of 27 patients with end-stage DSRCT, 10 (37%) had tumors that were positive for AR by IHC (35). Among the 10 AR-positive patients, 6 had been treated with combined androgen blockade (CAB). Three responded, with either stable disease or a reduction in tumor burden, though these responses were not durable, lasting only 3-4 months. The patients who responded to CAB had normal testosterone levels, whereas the patients who did not respond had castrate levels of testosterone, which could explain the lack of efficacy of CAB (35). A subsequent study found that AR-positive tumors were enriched for markers of stemness, which could also partially explain why the response to CAB in AR-positive patients with normal testosterone levels was short-lived (36).

In addition to the targets above, numerous other potential targets have been identified and are in various stages of pre-clinical investigation (**Table 2**). These include pathways common to many types of cancer (DNA damage-response pathway, epithelial-mesenchymal transition) as well as receptors that have been identified as overexpressed or activated specifically in DSRCT as targets of the EWS-WT1 transcription factor (LRRC15, NTRK3).

### Targets for Immunotherapy

A suitable target for immunotherapy must be expressed consistently on tumor cells but be restricted in its normal tissue expression to prevent on-target, off-tumor toxicity. Several appropriate targets have been identified in DSRCT. B7-H3 (targeted by the monoclonal antibody 8H9) is expressed in almost all DSRCT (35/37 tumors in one study (37), 44/46 in another) (38). It is tightly regulated by microRNA-29; despite universal transcription in most normal tissues, protein expression is highly restricted (39). Its hepatic expression explained the liver sequestration after intravenous injection, forcing its clinical development into compartmental administrations (NCT00582608). A clinical trial using intraperitoneal compartmental RIT using ^131^I-8H9 in DSRCT is ongoing (NCT01099644). Two studies have found GD2 expression in DSRCT, albeit to varying degrees (32/36 in one study (38), 2/20 in another.) (40) A T-cell engaging bispecific antibody (BsAb) trial is currently open for GD2(+) DSRCT (NCT03860207). EGFR, which is frequently mutated or overexpressed in cancer, was found to be amplified in 2/10 (20%) DSRCT tumors (41), and an EGFR CAR T cell trial for children and young adults with refractory/recurrent solid tumors (including DSRCT) is currently underway (NCT03618381). Another phase I CAR T cell trial was carried out for patients with HER2-positive sarcomas and the one DSRCT patient included achieved stable disease for 14 months (42). In a study assessing expression of various markers in DSRCT, HER2 was found to be expressed in 7/18 tumors, albeit at a low level for the majority of the positive-staining tumors (43). The use of these targets for immunotherapy in DSRCT is discussed in more detail below.

## Immunotherapy and Radioimmunotherapy for DSRCT

Although there has been a relatively recent resurgence in interest in cancer immunotherapy, the practice of using immunomodulatory agents to activate an anti-tumor immune response is over a hundred years old (44). Immunotherapy holds the potential for durable responses and even cures of metastatic disease. Immune cells activated against the tumor can seek out and destroy micrometastases and prevent recurrence. This type of treatment is particularly useful in DSRCT because of the extensive intraperitoneal seeding that typically has occurred at the time of diagnosis. While it is practically impossible to surgically remove every tumor cell when there are often dozens or hundreds of tumor implants on the peritoneum, removing all visible tumors surgically and following up with consolidation treatment to “clean up” leftover tumor cells is a viable strategy already in use for DSRCT in the form of HIPEC and abdominal radiation (14, 15, 17).

Another way in which immunotherapy is particularly well-suited for DSRCT and other cancers that affect mainly children and young adults is the lack of long-term adverse effects, or at least side effects that do not overlap with classic genotoxic chemoradiotherapy. Because these patients are treated early in life, any lasting adverse effects of treatment have the potential to impact them for decades. Furthermore, some treatments, including cranial radiation and intrathecal chemotherapy used to treat CNS metastases, have the potential to cause cognitive deficits and endocrinopathies (45). Although the wide-spread use of immunotherapy in children and young adults is relatively new, it also appears to be generally safe, though long-term follow-up will be required to identify any late adverse effects.

Because of these enormous potential benefits, various types of immunotherapy are being investigated for DSRCT. A summary of current clinical trials involving immunotherapy for DSRCT, and their molecular targets can be found in **Table 3**. Cellular targets that have been used for immunotherapy in other types of cancer have been identified on DSRCT tumors. For example, GD2 is reportedly expressed on the surface of DSRCT and is the target of a clinical trial (NCT00445965) employing the radionuclide-conjugated anti-GD2 antibody ^131^I-3F8 for patients with DSRCT and other tumors that either spread to or originate in the CNS. Neuroblastoma cells uniformly express GD2 and the use of monoclonal antibody therapy in high-risk neuroblastoma has been particularly encouraging (46).

**Table 3:** Previous and current clinical trials using immunotherapy to treat DSRCT.

Trial	Phase	Therapy	Type	Target	Status	Study term	Actual enrollment
NCT00043979	2	Allogenic hematopoietic stem cell transplant	STC	n/a	Completed	09/2002 – 12/2011	60
NCT00089245	1	^131^I-8H9	RIT	B7-H3	Active, not recruiting	07/2004 – 07/2021	120*
NCT00445965	2	^131^I-3F8	RIT	GD2	Active, not recruiting	01/2006 – 01/2022	78
NCT00562380	1	AMG-479	mAb	IGF-1R	Completed	04/2010 – 06/2010	64*
NCT00720174	1	Cixutumumab	mAb	IGF-1R	Completed	06/2008 – 01/2013	30
NCT01099644	1	^131^I-8H9	RIT	B7-H3	Active, not recruiting	04/2010 – 09/2021	54
NCT02982486	2	Nivolumab + ipilimumab	ICI	PD-1/CTLA-4	Unknown	12/2017 – 12/2020	60*
NCT02982941	1	Enoblituzumab	mAb	B7-H3	Completed	12/2016 – 05/2019	25
NCT03618381	1	EGFR806 CAR T Cells	CAR T	EGFR	Recruiting	06/2019 – 06/2038	36*
NCT03860207	1/2	Hu3F8-BsAb	BsAb	GD2	Recruiting	02/2019 – 02/2022	30*
NCT04022213	2	^131^I-8H9	RIT	B7-H3	Recruiting	07/2019 – 07/2024	55*
NCT04483778	1	B7-H3 CAR T cells	CAR T	B7-H3	Recruiting	07/2020 – 12/2040	68*
NCT04530487	2	Allogenic hematopoietic stem cell transplant	SCT	n/a	Recruiting	08/2020 – 05/2025	40*
NCT04897321	1	B7-H3 CAR T cells	CAR T	B7-H3	Not yet recruiting	04/2022 – 03/2027	32*

*For ongoing studies the estimated enrollment is provided in lieu of actual enrollment. STC, stem cell transplant; RIT, radioimmunotherapy; mAb, monoclonal antibody; ICI, immune checkpoint inhibitor; CAR T, chimeric antigen receptor T cell; BsAb, bispecific antibody.

### Antibody-Based Therapies

Several types of antibody-based therapies are used in the treatment of cancer, all of which rely on the specificity of an antibody-antigen interaction and require an appropriate tumor antigen to be identified that is expressed highly in tumor tissue and not in normal tissue. The most basic antibodies are monoclonal antibodies directed against a tumor antigen and designed to engage effectors cells through their Fc receptors, to mediate antibody-dependent, cell-mediated cytotoxicity (ADCC) either by natural killer cells (NK-ADCC) or by myeloid cells such as neutrophils and macrophages (myeloid-ADCC). In some tumors (e.g. neuroblastoma), complement-mediated cytotoxicity (CDC) can also play a role in the anti-tumor response. Enoblituzumab specific for B7-H3, is being tested in a phase I clinical trial in children and young adults with B7-H3-expressing relapsed/refractory solid tumors including DSRCT (NCT02982941). Enoblituzumab is also being tested in combination with checkpoint inhibitors pembrolizumab (NCT02475213) and ipilimumab (NCT02381314) among patients with B7-H3-expressing tumors.

There is also an ongoing trial to test the efficacy of a T cell-engaging BsAb in patients with advanced GD2+ tumors including DSRCT (NCT03860207). These BsAb are wide-ranging in structure, with one or more domains designed to engage T cells (usually targeting CD3) and one or more domains specific for a tumor antigen and serve as a sort of link between T cells and tumor cells, turning polyclonal T cells into specific killers. The advantage of BsAb over traditional antibody therapy is that they can initiate a robust T cell response against tumors at comparatively very low antibody concentrations and low target density. So far just one BsAb, blinatumomab, which targets CD3 and CD19, has been approved for use in cancer, in this case for ALL (47). Many others are in phase I-II trials, but only in adults (48).

Additionally, tumor antigen-specific antibodies can be used as vehicles to deliver radionuclide conjugates selectively to tumors in a process called radioimmunotherapy (RIT). This type of therapy has been successful clinically and two drugs have so far been approved for non-Hodgkin lymphoma (49). Currently, two radionuclide-conjugated antibodies are in trials for DSRCT: ^131^I-3F8, directed against GD2, which has previously been tested in medulloblastoma (50) and as a diagnostic tool in neuroblastoma (51), and ^131^I-8H9, directed against B7-H3, which has been previously tested in neuroblastoma (52). A recent Phase I trial (NCT01099644) of intraperitoneal ^131^I-8H9 in 48 DSRCT patients and four patients with other B7-H3-positive sarcomas found the treatment to be safe and well-tolerated with no dose-limiting toxicities (53). A Phase II trial for DSRCT is now accruing patients (NCT04022213).

### CAR T Cells

In addition to using bi-specific antibodies to direct T cells to tumors, it is also possible to redirect T cells by genetically engineering them to express receptors specific for more classic antibody targets, i.e. not T cell receptor (TCR) peptide-MCH targets. These engineered cells are termed chimeric antigen receptor (CAR) T cells. In the past two years, two CAR T cell therapies have been approved by the FDA for treating CD19-expressing cancers based on clinical trials that showed they can induce complete responses in a significant proportion of patients (54, 55). Although current success with CAR T cell therapy has been largely limited to liquid tumors, clinical trials for solid tumors, including DSRCT and other sarcomas, are ongoing (56). HER2-CAR T cells were used in a phase I/II trial of sarcoma patients (including one with DSRCT) because evidence shows that HER2 is expressed at low levels by many types of sarcoma, including DSRCT (43, 57). While this low level of expression precludes immunotherapy with IgG monoclonal antibodies, CAR T cell therapy could be effective (57). This trial showed that HER2-CAR T cells delivered at low doses were safe, tolerable, and effective in maintaining stable disease in a subset of patients, with one DSRCT patient stable for >14 months (42). Phase I trials testing CAR T cells targeting EGFR (NCT03618381) and B7-H3 (NCT04483778, NCT04897321) are currently underway for patients with solid tumors including DSRCT.

### Cancer Vaccines

It has been proposed that chromosomal translocation products can serve as tumor-specific antigens in sarcomas including DSRCT (58). This occurs when mutant proteins are processed by proteasomes and displayed as peptides on MHC molecules on the surface of tumor cells, as was demonstrated with mutant p53 (59, 60). A 9-amino acid epitope from the EWS-WT1 fusion protein has been described that binds to HLA-A3, providing the potential target for cytotoxic T cells (61). In one study among sarcoma patients, 2 patients with DSRCT were treated with autologous lymphocytes, tumor-pulsed dendritic cells and IL-7; unfortunately, neither had any measurable response (62). In another WT1 peptide vaccine trial among a group of 20 children with glioma, rhabdomyosarcoma, neuroblastoma, osteosarcoma, and clear cell sarcoma of the kidney (63), WT1-specific immune response was demonstrated in 4/18 (22%) evaluable patients (63). Although no DSRCT patients were included in this study, these results may provide a rationale for using peptide vaccines in DSRCT and other tumors with chromosomal translocation products that contain WT1.

### Hematopoietic Stem Cell Transplantation

Allogenic stem cell transplants (SCT) have been transformative in the treatment of hematological cancers. Although intense pre-transplant chemotherapy drastically reduces tumor burden, it rarely eliminates all tumor cells from the marrow compartment (64). Rather, a graft-*versus*-tumor effect is probably responsible for long term tumor control (65, 66). In this way, SCT is a form of cancer immunotherapy. Allogenic SCT has been shown to increase survival in advanced DSRCT (11.4 months *vs*. 1.9 months) (19). Autologous SCT has also been used but does not appear to be effective in increasing survival or reducing recurrence (18, 67). Yet, one DSRCT patient treated with CD34+ selected peripheral blood stem cells (PBSC) following surgical resection and high-dose chemotherapy, has maintained remission for over 10 years (68).

### Checkpoint Inhibitors

The field of immunotherapy was revolutionized by the advent of immune checkpoint inhibitors, drugs meant to release the “brakes” on T cells to reboot or to recruit T cells in their anti-tumor immune responses. Drugs targeting CTLA4 (ipilimumab) and PD-1 (programmed cell death protein 1) (nivolumab and pembrolizumab) have proven effective in treating melanoma, and, most excitingly, many of the patients who respond are alive years after treatment, suggesting that their remission is durable (69). Unfortunately, DSRCT, like sarcomas in general, do not respond well to checkpoint inhibition (36, 70). Typical among pediatric cancers driven by gene fusion, DSRCT has low tumor mutation burden, and hence few neoepitopes and insufficient anti-tumor T cell clonal frequencies (71). Tumor mutation burden, which often also correlates with a low degree of lymphocyte infiltration, is a predictor of response to checkpoint inhibitors (72). In contrast to melanoma and other tumors that are responsive to checkpoint inhibition, DSRCT appears to be an immunologically “cold” tumor, that is to say it is not heavily populated by tumor infiltrating lymphocytes (TILs) (73). DSCRT has low or no expression of PD-1/PD-L1 (programmed death ligand 1), suggesting that signaling along this axis is uncommon (41, 74). Put simply, removing the “brakes” from T cells is only effective if the host already has T cells capable of recognizing tumor epitopes just waiting to be rebooted, or if the naive T cells can be recruited to go after neoepitopes. Despite their failure as monotherapies in DSRCT, they could still hold promise when combined with other forms of immunotherapy, such as CAR T cells, cancer vaccines, and BsAb (75–77).

### Challenges in Immunotherapy for DSRCT

Low mutation load is now a well-recognized Achilles heel for most solid tumors in children and adolescents/young adults (71). This partly explained the paucity of TILs, which is made worse by the upfront use of aggressive chemotherapy, adopted as the standard of care from the time of diagnosis (6, 78). While this chemotherapy often succeeds at reducing tumor burden, it also depletes the patient’s immune cells, which may lead to depletion of effector cells, and especially T cells, thereby reducing the efficacy of immunotherapies (78).

Another challenge lies in achieving sufficient concentrations of antibody or large proteins in the tumor. When delivered intravenously, it may be difficult to achieve therapeutic doses of antibodies without on-target, off-tumor toxicity and other adverse effects. One solution for this is to deliver the immunotherapeutic agent regionally or to a specific biological compartment (56). Currently, a clinical trial (NCT00445965) is underway to test the efficacy of intrathecal delivery of the radiolabeled antibody ^131^I-3F8 to DSRCT and other tumors that have spread to or originated in the brain. Another clinical trial (NCT04022213) is in progress to determine the efficacy of ^131^I-8H9 delivered intraperitoneally for DSRCT and other solid tumors which metastasize within the peritoneum. Regional delivery has also been tested in radioimmunotherapy of neuroblastoma that has spread to the CNS, and even CAR T cells in ovarian cancer (52, 56, 79). These delivery methods aim to allow the treatment to reach sufficient levels in and around the tumor while protecting other normal tissues from the deleterious effects of high concentrations of these immunotherapeutic agents. Given the peripatetic presence across all tissues, T cells have the capability to penetrate deep into tissues unlike antibodies. Hence BsAbs that arm T cells either *in vivo* or *ex vivo* may be able to overcome penetration hurdles.

Although speculative, regional or compartmental delivery of BsAb may reduce on-target, off-tumor effects, which can be life-threatening. The most common adverse effect experienced by patients in these T cell-based therapy is cytokine release syndrome, which occurs when large numbers of T cells become activated and release cytokines such as IFN-y, GM-CSF, IL-10, and IL-6 (80). This occurs because of immediate contact between active T cells with targets in the hematogenous compartment (e.g. CD19), or direct activation of T cells even before arriving at the tumor site. The effects of this “cytokine storm” range from mild to severe, and can cause fever, fatigue, pain, nausea, hypotension, and organ failure (80). One patient treated with 1 × 10^10^ HER2 CAR T cells experienced respiratory distress within minutes of the infusion and died 5 days later (81). It is thought that the CAR T cells localized to her lungs and became active upon recognition of low levels of HER2 expressed in lung tissue (81). However, patients have safely been treated with HER2 CAR T cells at much lower doses, including one DSRCT patient treated with 1 × 10^7^/m^2^ cells (42). Such life-threatening complications can potentially be avoided if the BsAbs are first administered into a “cold” compartment to bind to tumors before TILs arrive.

Finally, it is well known that tumors evolve resistance to treatments by downregulating expression of target molecules, and by upregulating expression of immune checkpoint molecules. In the context of immunotherapy, this is called immune escape (82). It has been demonstrated that tumors upregulate PD-1 and CD47 in response to treatment with BsAbs (77, 83). It has also been shown that tumors are capable of downregulating the targets of CARs, evidenced by the fact that 11% of B-cell malignancy patients who initially responded to treatment with CD19 CAR T cells eventually relapsed with CD19- tumors (84). One potential strategy to combat this phenomenon is to develop treatments that target multiple tumor antigens, including bispecific CAR T cells and T cell engaging antibodies with more than one tumor-targeting domain (82).

## Discussion

Overall cancer death rates have steadily declined over the past several decades in the United States, owing to prevention, early detection, and advances in treatment (85). Deaths from childhood cancers have also declined, though this is primarily due to the availability of better treatments, since childhood cancers (which are rarely caused by infection or exposure to carcinogens) can only rarely be prevented (86, 87). Furthermore, while the adult cancer death rate has been improved by efforts to regularly screen at-risk populations (through cervical cancer screening, mammograms, and colonoscopies, for example), for childhood cancers including DSRCT it is not feasible to implement any screening programs because no at-risk populations have been identified and no suitable screening tests exist to implement on a population-wide scale (88–90). For these reasons, efforts to reduce deaths from DSRCT and other childhood cancers can only be focused on improving treatments. While some novel approaches, such as the use of HIPEC, could delay progression, few have yielded few long-term DSRCT survivors who can be considered cured (6, 14). Immunotherapy and radioimmunotherapy are attractive options for DSRCT because they have the potential to produce durable responses without long-term side effects.

So far most of the reports on immunotherapy success in DSRCT are anecdotal. However, it is useful to learn from the successful implementation of immunotherapy in other pediatric solid tumors, like high-risk metastatic neuroblastoma which was incurable three decades ago. As mentioned previously, GD2-targeted immunotherapy has revolutionized treatment of high-risk neuroblastoma even when recurrent (46). Beginning with murine monoclonal antibodies, anti-GD2 treatment has evolved into chimeric and humanized monoclonal antibodies, and trials are underway testing CAR T cells (NCT03294954), an antibody vaccine(NCT00911560), and BsAb therapy (NCT03860207) (46). Though decades behind neuroblastoma, DSRCT could benefit from similar types of immunotherapies.

While DSRCT and other immunologically “cold” tumors don’t have *de novo* anti-tumor immune responses, passive immunotherapy, such as monoclonal antibodies, may still be effective. The success of monoclonal antibody therapy in neuroblastoma and leukemia, even in patients who are immunocompromised by prior chemoradiotherapy, suggests that these passive immunotherapeutic approaches could be successfully implemented even after receiving dose-intensive therapies among DSRCT patients. In fact, a monoclonal antibody against B7-H3 (NCT02982941) and two radionuclide-labeled monoclonal antibodies against B7-H3 (NCT04022213) and GD2 (NCT00445965) are currently being tested in clinical trials open to patients with DSRCT.

Immunotherapy may also be made more efficacious for DSRCT by compartmental delivery to the peritoneum. While DSRCT is often advanced at diagnosis, in a substantial proportion of patients their disease is contained within the peritoneum. By delivering the immunotherapeutic agent intraperitoneally, the concentration of the agent in and around the tumor could be substantially higher compared to systemic delivery. The likelihood of adverse on-target, off-tumor effects would also likely be reduced for tissues expressing the target but located outside the peritoneal cavity. In this way, the impact of the agent on the tumors would be maximized while the impact of the agent on normal tissue would be minimized. Intraperitoneal delivery of CAR T cells has proven superior to systemic delivery in an animal model of metastatic peritoneal colorectal cancer (91) and this strategy is being implemented in clinical trials for ovarian cancer (NCT02498912), peritoneal mesothelioma (NCT03608618) and advanced gastric cancer (NCT03563326) with peritoneal spread. When DSRCT does spread beyond the peritoneum, it often metastasizes to the CNS, which is walled off by the blood brain barrier from most treatments that are delivered parenterally. To reach these tumors treatment must be delivered intrathecally or intraparenchymally using catheters connected to an Ommaya reservoir. A clinical trial testing intrathecal ^131^I-3F8 (NCT00445965) is ongoing for patients with CNS tumors, including metastatic DSRCT.

Another challenge to consider which may inform the future direction of immunotherapy in DSRCT is antigen heterogeneity and antigen loss, a process in which tumors lose expression of antigens targeted by immunotherapy and thereby “escape” immune surveillance. This is now a well-known mechanism of resistance in CD19 CAR T cells, where recurrent leukemia cells no longer express CD19 following treatment. A proposed solution for this problem is to target two antigens instead of one, since the probability of two distinct mutations arising in the tumor that would downregulate both targets and allow for antigen escape during treatment is statistically unlikely (92). Since DSRCT expresses several tumor-associated targets, they should be suitable for this dual targeting approach. Targeting multiple antigens would also increase efficacy of immunotherapy in tumors where the expression of targets is heterogenous, where no single target is expressed uniformly on every tumor cell. Radionuclide-conjugated antibodies offer a similar benefit, as they can kill not only the tumor cells that express their antibody targets but other tumor cells in the vicinity. This killing is achieved by way of cross-firing during radioactive decay by high energy particles such as electrons and positrons that travel millimeter distances, and alpha particles that travel micron distances. By combining antibody formats that target multiple targets with novel radioimmunotherapy platforms, the potential exists for designing a radioimmunotherapy strategy for DSRCT, which is well-known to be radiation-responsive.

In addition to the scientific challenges confronting immunotherapy approaches, it is important to take into consideration the logistical hurdles, including the cost of these therapies and the accessibility for the general public world-wide. These issues have surfaced quickly in the field of CAR T cells, which must be tailor-made for each patient using *ex vivo* T cell expansion and viral transduction. In the case of Kymriah and Yescarta, the two FDA-approved CAR T cell therapies, the drug cost is currently $475,000 and $373,000 per patient, respectively (93). These prices make them inaccessible for many patients, even those in developed countries. In addition to the cost, the treatment can take several weeks to prepare for each individual patient. Although these therapies have undoubtedly saved lives, the fact remains that they are time-consuming and expensive to produce. While antibody therapy is less personalized and therefore does not have to be tailored made for each patient, it is still expensive in the current market. In the United States, a course of treatment with dinutuximab, an anti-GD2 monoclonal antibody, for example, can cost upwards of $150,000 for the drug only, and the only BsAb approved for cancer, blinatumomab, costs over $170,000 per year. The cost of these revolutionary treatments needs to be considered when evaluating the success of immunotherapy, considering that patients cannot benefit from drugs they cannot access.

Until recently, the improvements in survival for DSRCT have been incremental, owing mostly to the use of multi-agent chemotherapy. These combinations of drugs are highly toxic, have debilitating side effects, and only serve to prolong survival by a few months or years, at best. Today, there are numerous ongoing trials of various types of immunotherapy for DSRCT patients, including a BsAb, radionuclide-conjugated antibodies, a monoclonal antibody, CAR T cells, and checkpoint inhibitors (**Table 3**). New clinical studies aiming to collate information on clinical attributes (NCT04690374) and immune characteristics (NCT03967834) of patients with DSRCT will improve our knowledge this rare disease. Immunotherapy offers the promise of eradicating chemotherapy-resistant, microscopic tumors that cause relapse and eventually death in a majority of DSRCT patients, without the same adverse long-term effects encountered by genotoxic therapies. It is instructive to look at the successes of immunotherapy in other diseases, such as neuroblastoma and leukemia, which have seen children with relapsed tumors cured for the first time by antibody therapy and CAR T cells. At the same time, it is important to learn from the challenges experienced in the development and evolution of these treatments so that they may be implemented most efficiently and effectively in DSRCT.

## Author Contributions

N-KC and ME-C both wrote and edited this manuscript. All authors contributed to the article and approved the submitted version.

## Funding

The authors acknowledge support of the NCI Cancer Center Support Grant P30 CA008748, NCI Predoctoral to Postdoctoral Fellow Transition Award K00 CA223062, and support from the following donors and organizations: Runway Heroes, Renaissance Charitable Foundation, Tay-Bandz, Inc., The Steven Vanover Foundation, and Brenda D. Vanover.

## Conflict of Interest

N-KC reports receiving commercial research grants from Y-mAbs Therapeutics and Abpro-Labs Inc.; holding ownership interest/equity/options in Y-mAbs Therapeutics Inc., and in Abpro-Labs, and owning stock options in Eureka Therapeutics. N-KC is the inventor of pending and issued patents filed by MSK, including hu3F8 and 8H9 licensed to Y-mAbs Therapeutics, beta-glucan to Biotec Pharmacon, and HER2 bispecific antibody to Abpro-labs. N-KC is an advisory board member for Abpro-Labs and Eureka Therapeutics.

The remaining author declares that the research was conducted in the absence of any commercial or financial relationships that could be construed as a potential conflict of interest.

## Publisher’s Note

All claims expressed in this article are solely those of the authors and do not necessarily represent those of their affiliated organizations, or those of the publisher, the editors and the reviewers. Any product that may be evaluated in this article, or claim that may be made by its manufacturer, is not guaranteed or endorsed by the publisher.
